# Electroacoustic Stimulation: Now and into the Future

**DOI:** 10.1155/2014/350504

**Published:** 2014-09-04

**Authors:** S. Irving, L. Gillespie, R. Richardson, D. Rowe, J. B. Fallon, A. K. Wise

**Affiliations:** ^1^Bionics Institute, Melbourne, VIC 3002, Australia; ^2^Department of Psychology, University of Melbourne, Melbourne, VIC 3010, Australia; ^3^Department of Otolaryngology, University of Melbourne, Melbourne, VIC 3010, Australia; ^4^Department of Medical Bionics, University of Melbourne, Melbourne, VIC 3010, Australia

## Abstract

Cochlear implants have provided hearing to hundreds of thousands of profoundly deaf people around the world. Recently, the eligibility criteria for cochlear implantation have been relaxed to include individuals who have some useful residual hearing. These recipients receive inputs from both electric and acoustic stimulation (EAS). Implant recipients who can combine these hearing modalities demonstrate pronounced benefit in speech perception, listening in background noise, and music appreciation over implant recipients that rely on electrical stimulation alone. The mechanisms bestowing this benefit are unknown, but it is likely that interaction of the electric and acoustic signals in the auditory pathway plays a role. Protection of residual hearing both during and following cochlear implantation is critical for EAS. A number of surgical refinements have been implemented to protect residual hearing, and the development of hearing-protective drug and gene therapies is promising for EAS recipients. This review outlines the current field of EAS, with a focus on interactions that are observed between these modalities in animal models. It also outlines current trends in EAS surgery and gives an overview of the drug and gene therapies that are clinically translatable and may one day provide protection of residual hearing for cochlear implant recipients.

## 1. Introduction

Cochlear implants have successfully provided hearing to over three hundred thousand hearing impaired people worldwide [1]. Traditionally, implantation was carried out only in recipients with profound hearing loss, but improvements in technology and sound processing techniques, coupled with the recent relaxation of the eligibility criteria, has led to more implantees with some degree of low-frequency residual hearing [2, 3]. These recipients receive both electrical stimulation from their cochlear implant and acoustic stimulation via their residual hearing (electroacoustic stimulation; EAS).

**Figure 1:**
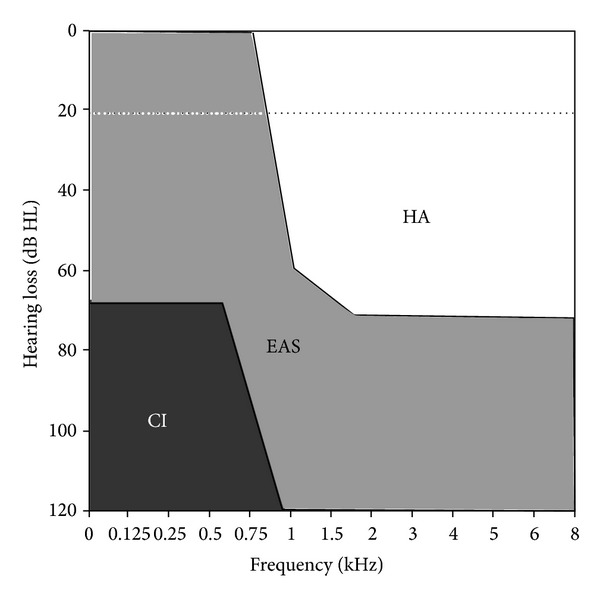
Typical hearing ranges (in dB HL) showing candidature for hearing aids (HA), electroacoustic stimulation (EAS), and cochlear implant use alone (CI).

The typical EAS recipient is an adult who has lost high frequency hearing postlingually, whilst maintaining usable hearing in the low frequencies, creating a so-called ‘ski-slope' hearing loss (Figure 1, [4]). It is likely that the number of undiagnosed partial hearing children is larger than typically accepted [5] and, as such, the number of children using EAS is also likely to rise. Furthermore, the prevalence of high frequency hearing loss is increasing worldwide due to growing environmental and recreational noise and an ageing population. As a result, it is likely that in the future, more cochlear implant recipients will maintain some degree of usable hearing.

EAS recipients display substantial benefits in hearing performance compared to profoundly deaf recipients who rely on electrical stimulation alone in pitch perception [6], speech perception [2, 4, 7–10], listening in background noise [11–14], and music appreciation [8, 15]. Recent reviews have discussed the clinical benefits of EAS over the use of electrical stimulation alone [6, 16], as well as the fitting ranges, outcomes, and clinical practice in EAS [10], and the reader is directed there for more information about these aspects of EAS. Despite the clear clinical benefits, little is known of the mechanisms that contribute towards them, although it is thought that the interactions between the electrical and acoustic stimuli may play a role therein. In order to explore the neural mechanisms of EAS integration, as well as optimising clinical applications, the use of animal models is essential. To date, surprisingly little research has been carried out into EAS using such models, and fewer still have used animal models with hearing thresholds that reflect those seen in the clinic (with the exception of [17]).

Reports of recipients suffering immediate or delayed loss of low frequency residual hearing following cochlear implantation [4, 6, 18] are concerning: the use of short, atraumatic electrode arrays to minimise cochlear damage, such as the Cochlear Hybrid-S or the Med-EL Flex arrays [2, 18], potentially leaves the more apical regions of the cochlea unstimulated. If cochlear implantation causes residual hearing to deteriorate, these recipients could lose the benefits bestowed by EAS but also do not have the optimum electrical stimulation provided by longer electrode arrays and may therefore require reimplantation [19]. Further investigation into the mechanisms of improved hearing is crucial for optimisation of EAS processing strategies, but also to find ways to minimise the negative effects of cochlear implantation on residual hearing to protect hair cell and spiral ganglion neuron (SGN) function.

The maintenance of existing hearing is critical for EAS, and research has focused on numerous factors that could protect hair cells and SGNs after hearing loss and during cochlear implantation including neurotrophic factors, anti-inflammatory steroidal drugs, antiapoptotic agents, or a combination of these. The means of locally delivering these agents have also been well researched, in particular the challenge of protecting SGNs after hearing loss due to the need for continuous exposure to neurotrophins for long-term SGN survival [20, 21], as survival has not been reported to last beyond 2 weeks after the cessation of neurotrophin delivery [22, 23]. Hence, single-intervention approaches with long-term outcomes such as gene or cell-based therapies are of particular interest.

This review focuses on the current preclinical EAS research, as well as discussing potential therapies that may be combined with electrical stimulation to maintain optimal cochlear and neural health in cochlear implant users with residual hearing. In particular, we will focus upon the interactions and integration between the two stimulation modalities at a neural level, from the cochlea to the auditory cortex, as well as discussing the current practices to reduce loss of residual hearing. Furthermore, we will discuss the potential of gene therapy to provide a long-term or constant supply of neurotrophins from a single intervention to promote SGN survival (and therefore residual hearing) after partial hearing loss, with particular emphasis on the use of viral vectors for cell specific gene expression and discussion of clinical safety.

## 2. Electroacoustic Stimulation

One of the main areas of research into EAS focuses on the interactions between the responses to the electric and acoustic stimuli. While the clinical evidence indicates improved performance with EAS, it is possible that the stimuli can also effectively mask one another, reducing the quality of the incoming signal. If this were the case, additional clinical benefit may be achieved by segregating the signals either temporally or spatially (with regard to the intracochlear regions that each stimulus type activates) so that masking is minimised. This section presents an overview of the known interactions between electric and acoustic hearing, as well as discussing plastic changes that occur in the brain due to combined stimulation.

### 2.1. Physiological Interactions between Acoustic and Electric Stimulation

It is important to note that the majority of studies investigating EAS interactions to date have been carried out in normal-hearing animals fitted with intra- or extracochlear stimulating electrodes [24–26]. Although these models give an indication of the interactions in healthy cochlear conditions, they do not necessarily reflect the listening conditions seen in EAS recipients, which will have degraded cochlear processing. Nevertheless, the increasing trend to implant recipients with more and more residual hearing is likely to cause an increase in the number of recipients in which a near normal cochlear region receives stimulation from both electric and acoustic stimulation “overlap,” and these results are therefore of considerable interest.

#### 2.1.1. Interactions in the Normal Hearing Cochlea

**Figure 2:**
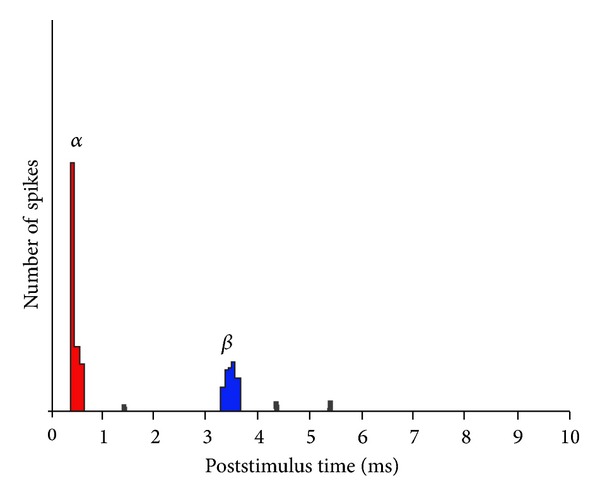
Stylised poststimulus time histogram showing examples of *α*- and *β*-responses recorded at the auditory nerve in response to electrical stimulation. The *α*-response is from direct activation of the auditory nerve and the *β*-responses are caused by electrophonic activation.

The first report of the effects of EAS is from the level of the cochlea in the doctoral dissertation of Moxon [27]. Using auditory nerve recordings, Moxon demonstrated that electric stimulation at low current levels within the normal hearing cochlea generated hair cell-mediated response (known as “electrophonic” responses; the *β*-component of the auditory nerve response). Higher current levels produced a short latency *α*-component which results from direct stimulation of the auditory neurons (Figure 2). This suggests that, in a cochlea with residual hair cells, electric stimulation activates the auditory nerve through a dual pathway: via the SGNs directly and indirectly through “normal” transduction of the electrically-generated displacement of the basilar membrane. Electrophonic effects on auditory function are discussed below.

Recordings of compound action potentials (CAPs) under different stimulation combinations have made up the majority of the electrophysiological studies investigating EAS. This work has notably used masking paradigms, including forward masking of an acoustic signal by electric stimulation, forward masking of an electric signal by an acoustic signal and simultaneous EAS.

Experiments in normal hearing animals show that acoustically-evoked CAPs are suppressed by a preceding electric pulse train presented at the base of the cochlea, with the strongest suppression occurring for responses to low intensity, high frequency acoustic stimuli that were masked by high current levels [24]. Suppression of low frequency acoustic stimuli only occurred for higher current levels, likely due to current spread to the apical region of the cochlea, and did not occur in all animals. This suggests that EAS interactions require a physical overlap between the hair cells and the stimulating current. Hence, it is likely that interactions seen in normal hearing experimental animals implanted with cochlear implants would be larger than those seen in partial hearing situations. The observed suppression is not solely due to the refractoriness of the nerve after the masking stimulus, as the latency of the suppressive effect is longer than is seen in spontaneous firing and may be due to suppressive effects from the hair cell (see discussion on electrophonics, below).

Further studies have investigated the effects of an acoustic masker on the electrically-evoked CAP (ECAP) and have shown that broadband noise can decrease both the ECAP amplitude and firing synchrony [28–30], resulting in increased electrical thresholds. This effect was seen both during and after the masking noise was presented (forward- and simultaneous masking), although it was largest for simultaneous masking. Electrical thresholds returned to premasking levels between 100–200 ms post masker offset. Masking was particularly prominent at low electrical pulse rates (>3 ms interpulse interval) but absent at higher rates [29].

The role of background activity in EAS interactions was demonstrated by Miller et al. [30], who showed that adding an acoustic noise to an electric pulse stimulus increased temporal variability of spikes in the auditory neurons (“jitter”), but that in the 20 ms period following the offset of an acoustic masker, electrical responses showed a decrease in jitter. This finding was limited to nerve fibres that exhibited high levels of spontaneous activity, suggesting that the acoustic signal was able to vary firing synchrony across the different auditory nerve fibres. Miller and colleagues [30] have further shown that simultaneous EAS caused an increase in spike rate in auditory nerve fibres compared to electric stimulation alone, corroborating findings by Von Ilberg et al. [25]. Spike rate increased with current level of the electric stimulus, but an increase in spike rate seen in EAS was not equal to the sum of the respective electric and acoustic firing rates. Conversely, the temporal jitter did not vary between EAS and electric stimulation alone conditions.

The tuning of auditory nerve fibres under EAS stimulation has only been investigated in one study [25], which found that EAS did not alter the characteristic frequency (the frequency with the lowest threshold) of the auditory nerve fibres, either acutely or after chronic stimulation. Tuning curves in sharply tuned fibres were broader under combined EAS, but no change was seen in fibres that were already broadly tuned. All characteristics returned to normal after cessation of electrical stimulation.

The dependence of the electric and acoustic masking upon the relative temporal position of the stimuli is of clinical relevance because the latter depends upon a number of factors: (a) the frequency of the stimulus, (b) the relative delay introduced by the speech processor, and (c) the power spectrum of the stimulus.

**Figure 3:**
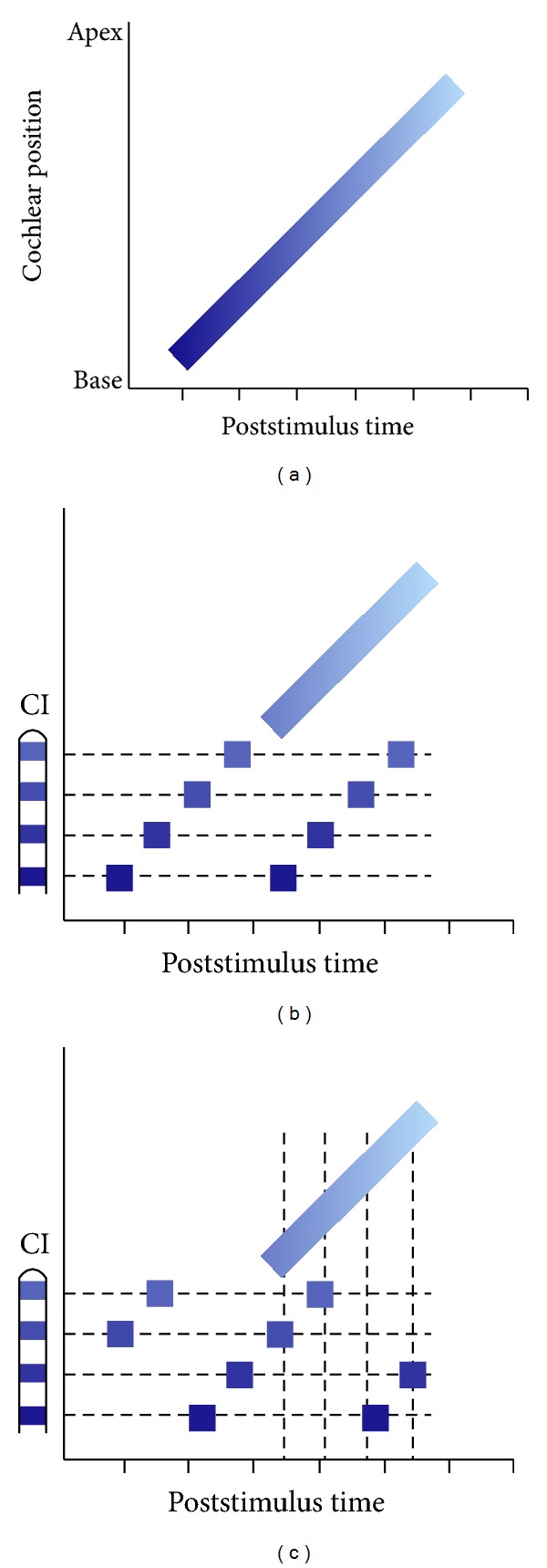
Illustration of potential interference between electric and acoustic stimulation to the same stimulus. (a) represents the normal hearing case, where the travelling wave causes the base of the cochlea to be activated before the apex in a systematic manner. Colour indicates stimulation at a particular cochlear location. (b) and (c) show an EAS cochlea (cochlear implant represented on the left), where the round robin processing strategy causes simultaneous activation of two distinct regions of the cochlea for electric and acoustic stimuli. In (b), the round robin sequence begins at the most basal electrode, whereas (c) shows the stimulation occurring first to the second most apical electrode. These panels represent the response to the same stimulus but depict how the location of the stimulating electrode within the round robin sequence can cause different temporal electrode/cochlear position combinations for the same external stimulus.

Stimulus frequency will affect relative timing between the two stimulus modalities due to the travelling wave [31], where apical cochlear regions stimulated by lower frequencies are reached later than the more basal, high-frequency regions, a delay that can vary between 1 and 10 ms from base to apex [32]. A further degree of temporal variability is derived from the processing carried out by the speech processor and the “round robin” stimulation paradigm used in most stimulation strategies, where electrodes are stimulated sequentially (Figure 3. [33]) and therefore depends upon the stimulus, the processing strategy used and the length of the electrode array. It is entirely possible in a region of overlapping electrical stimulation and residual hearing that the same acoustic stimulus could lead to sequential forward masking of both the acoustic response and the electric response by the other. It is currently unknown how such a stimulus is encoded at the level of the auditory nerve and higher up the auditory pathway.

#### 2.1.2. Electrophonic Suppression of Auditory Nerve Responses

The electrically-induced basilar membrane motion that gives rise to electrophonic responses was thought to be generated by the electromotile properties of the outer hair cells (OHCs; [16, 34, 35]). However, more recent research investigating the role of the OHCs in electrophonic generation has shown that destruction of OHCs does not abolish the electrophonic component of the CAP [36]. Therefore, as long as some IHCs remain in EAS recipients, there is the opportunity for electrophonics to occur (and at lower current levels, they may be amplified by OHC activation [36]). Stronks et al. [36] also showed that longer electrical pulse widths cause greater electrophonics and suggested that in order to reduce their inhibitory effects, short electrical pulses should be used clinically. Nevertheless, it is currently unclear as to whether these interactions are undesirable from a perceptual perspective and further investigation should aim to answer this question.

#### 2.1.3. Interactions in the Partially Deaf Cochlea

Although most studies investigating EAS in animal models to date have used normal-hearing animals, there has been a recent increase in the number of published studies that aim to emulate a clinically relevant partial hearing loss [28, 37], and we have recently described a model for chronic cochlear implant use in a partial hearing animal model that is directly relevant to EAS [17]. The use of such partial hearing models in EAS research is essential to enable more clinically-relevant questions to be addressed. For example, Stronks et al. [38] observed a significant decrease in CAP suppression by electrical forward masking in guinea pigs that had been partially deafened with a combination of furosemide and kanamycin compared to normal hearing controls. This suggests that partial hearing cochleae implanted with intracochlear electrodesmay notdisplay interactions at the level of the auditory nerve, even when using high current levels to stimulate the apical regions with intact hearing. This finding is important as it suggests that the interactions described in normal hearing animals above may not occur in clinical populations, who typically have a high frequency hearing loss that does not overlap with the cochlear region that is stimulated by the cochlear implant and may therefore have little bearing on “real life” EAS responses. Further research is required to investigate these interactions in partial hearing EAS situations.

A further confound between clinical populations and animal models of partial hearing loss comes in the form of the fitted device. For recipients with residual hearing, it is common for combination devices that couple acoustic amplification with electrical stimulation to be used in the implanted ear [39]. Hearing aids are unavailable for chronic use in animal models, and additional experimental benefits would be seen in mimicking hearing loss that matches that seen in recipients using amplified hearing in the lower frequency region.

#### 2.1.4. Evidence of Central EAS Interactions

EAS interactions at the level of the inferior colliculus (IC) in normal hearing animals have been investigated by Vollmer et al. [40]. As described for the auditory nerve (see above), forward masking of an acoustic stimulus by single biphasic electrical pulses caused a current-dependent decrease in IC responses to acoustic stimuli and resulted in elevated acoustic thresholds. Simultaneous electrical and acoustic presentation with a tone at the neuron's CF resulted in interactions that depended upon the relative levels of each of the two stimuli, with increasing suppression with electric masker level and decreased suppression with acoustic probe level (i.e. if the masker was at a higher level than the probe, then there was a greater suppressive interaction between the two). Simultaneous presentation of acoustic tones with electrical stimulation led to suppression of the electrically evoked response, which increased with increasing acoustic level. Overall, electrical stimulation at higher levels dominated the acoustic response and combined EAS resulted in increased spike rates, in agreement with findings in the auditory nerve [30].

### 2.2. Neural Plasticity Seen with Combined EAS

There is evidence that the speech recognition benefits that recipients experience with EAS develop over time [2], suggesting that the combined stimuli are causing plastic changes in the brain that enable the improved performance. Reiss et al. [41] demonstrated that the pitch percept provided by electrical stimulation at a particular cochlear location could change over time in EAS recipients compared to an acoustic reference. This shift could be as large as three octaves in some participants and generally caused the electric pitch to align with the frequency that was allocated to the electrode by the speech processor. The mechanisms of these plastic changes are, to date, unknown. Few studies have looked at the plastic effects of chronic EAS use at the physiological level, primarily due to a lack of suitable animal model (although see [17, 42]).

We have previously reported that chronic intracochlear electrical stimulation in cats with a high frequency hearing loss caused a decrease in the extent of primary auditory cortex that could be activated by acoustic stimulation [42]. Although characteristic frequency of the cortical neurons did not change with EAS, there were fewer neurons responsive to acoustic only stimulation, compared to electrical only and combined stimuli. As this study did not obtain recordings at the beginning of the stimulation period, it is impossible to determine whether these changes correspond to the pitch shifts reported by Reiss et al. [41], although it is apparent that plastic changes do occur in cases of EAS, warranting further examination to enable optimisation of clinical outcomes.

This section has outlined the physiological interactions between electric and acoustic stimulation, which are typically inhibitive for forward and simultaneous masking paradigms. However, there is currently little evidence of these interactions in clinically-relevant partial-hearing animals, although the plastic changes seen in chronically stimulated EAS animal models [41] could contribute to the success of EAS recipients. The use of partial-hearing models is of critical importance for the development and refining of EAS strategies and developing procedures to maintain residual hearing after chronic cochlear implantation. Regardless of the mechanisms involved in improved listening performance with EAS over electrical stimulation alone, the protection of any residual hearing is, by definition, vital to EAS. The following sections describe the potential causes of hearing loss associated with cochlear implantation, as well as discussing the procedures that can be undertaken to minimise loss of hearing during and after cochlear implantation.

## 3. Potential Causes of Hearing Loss following Cochlear Implantation

Critical to the success of EAS is the health of the cochlea and, in particular, the residual hearing available to the listener. It is well reported in the literature that the insertion and use of a cochlear implant can cause a loss of residual hearing in the stimulated ear. This section outlines potential causes for this loss, which can be surgical or histopathological.

Surgical factors are controlled by the implanting surgeon and include electrode selection, insertion route (cochleostomy or through the round window), insertion depth, and the use of atraumatic surgical techniques. Early traumas such as damage to the osseous spiral lamina, basilar membrane rupture, and lateral wall disruption have all been shown to have occurred during human cochlear implantation [43–45] and animal models of implantation [46, 47] and are likely to manifest as immediate hearing loss. The literature also suggests that implant surgeons have a standardised insertion technique appropriate to the electrode which is unrealistic, given the variations in cochlear anatomy, and could be a potential source of disparity. In addition, it is often assumed that hearing in the implant recipient is stable preoperatively, which is not always the case. As such, it is possible that implantation trauma accelerates the underlying cause of hearing loss and could further cloud outcome measures.

The cochlea's histopathological response to electrode insertion may also contribute to delayed or progressive hearing loss following cochlear implantation. Delayed effects include fibrotic changes around the electrode, new bone formation and foreign body reaction. There are a number of cochlear structures that can be affected by these changes after implantation, including the organ of Corti, the SGNs and their dendrites, and the stria vascularis along the lateral wall [43–45]. Altered electrode performance and hearing/speech outcomes do not always have an obvious causal relationship to damage of these delicate intracochlear structures and interpreting the role and impact of observed histopathological changes in hearing loss can be problematic. In addition, the role of cochlear mechanics and endolymphatic hydrops in affecting outcomes is unclear, and they remain possible contributing factors to the loss of residual hearing.

### 3.1. Surgical Factors in Loss of Residual Hearing

#### 3.1.1. Electrode Insertion Depth

Successful preservation of residual hearing and prevention of delayed hearing loss have generally been advocated by either surgical technique alone or use of a particular electrode design [8, 9, 48–51]. Gantz and Turner [48] reported that residual low frequency hearing was preserved in a group of 24 volunteers using either a 6 or 10 mm Iowa/Nucleus Hybrid Cochlear Implant, suggesting that a short electrode prevented any damage to the low frequency regions of the cochlea [8]. The authors reported good preservation of low frequency hearing in the long term (up to 20 years) with almost all subjects retaining their residual hearing. The perceived advantage behind the shortened electrode array is that it would cause less damage on insertion to the lateral wall of the cochlea at the basal turn and would not reach the low frequency areas at the apex of the cochlea. A more recent report by Woodson et al. [52] suggested that delayed hearing loss has occurred in some recipients receiving the Iowa/Cochlear Hybrid cochlear implant. They reported preservation of residual hearing (loss less than 30 dB of preoperative thresholds) in 91% of their subjects. This amount decreased to 75% by the end of the trial period suggesting a progression of hearing loss in some recipients.

Concerns over the potential loss of residual hearing are an important consideration for prospective recipients with significant residual hearing prior to implantation with a “hybrid” or shortened electrode. Given the potential for loss of residual hearing that is either due to the surgery, the biological response to the presence of the electrode array, or to progression of the underlying pathology, there is a potential benefit in having a longer electrode array with more electrodes within the cochlea that would allow for programming flexibility and pitch-matching similar to that used with current electrode arrays [53]. Were recipients to lose their residual hearing in the future, the cochlea would then have adequate coverage with a standard length array in the electrical stimulation only situation and obviate the need for reimplantation with a full length electrode at a later time as seen in some situations [54, 55]. The implantation of longer arrays in patients with residual hearing should be undertaken with caution, however, as a review by Boyd [56] suggests that deeper electrode insertion typically leads to greater cochlear injury. The use of longer electrodes may therefore increase the likelihood of loss of residual hearing compared to shorter electrode arrays, and this risk should be discussed with prospective patients.

#### 3.1.2. Soft Surgery

“Soft surgery,” a collection of techniques that would aid in the preservation of hearing following cochlear implantation, was first proposed as a concept by Lehnhardt in 1993 [57]. This protocol aims to minimise cochlear trauma by minimising the size of the cochleostomy, locating it at the level of the promontory to facilitate insertion into the scala tympani, maintaining an intact ossicular chain and not aspirating the perilymph [58]. Furthermore, histological studies have demonstrated less insertion trauma with round window insertions (i.e. without cochleostomy) [59–61] and reported excellent residual hearing preservation using round window insertions of partially inserted MED-EL electrodes. The idea that superior surgical technique (i.e. soft surgery) alone is enough to preserve residual hearing was challenged by Cohen [62] asserting that residual hearing is universally lost following implantation, irrespective of the implanted electrode and the technique of surgical insertion. Despite this assertion, many surgeons still strive to prevent hearing loss following implantation by adopting soft surgical techniques.

#### 3.1.3. Hypothermia

Safe hypothermic induction can be noninvasive by using cooling blankets and ice packs, or a more invasive route can involve safe infusion of large volumes of cold fluids [63]. Hypothermia is used clinically to promote neuronal survival in cardiac surgery and after cardiac arrest [63] by decreasing metabolic rate, reducing tissue oxygen consumption, depressing metabolic acidosis [64], suppressing calcium influx into neurons [65], and diminishing nitric oxide production [66]. Similar protective effects have been described during cochlear implantation, noise trauma and ischemic cochlear injury [67–71]. Further protective effects may arise from decreased glutamate release upon neuronal inflammation after trauma [72]. Despite these findings, the use of hypothermia during cochlear implant surgery has yet to become mainstream practice.

### 3.2. Histopathology in the Cochlea

#### 3.2.1. Cochlear Reaction to Implantation

Following implantation, the cochlea shows several pathological responses resulting from foreign body response to the electrode array. Apoptosis of auditory hair cells and SGNs [73, 74], inflammation resulting in fibrosis and osteogenesis [45, 75] and foreign body reaction [76] have all been reported. Rizer et al. [77] suggested that, following cochlear implantation, an inflammatory reaction occurred that ultimately resulted in residual hearing loss. A further suggestion was that the presence of the electrode and/or fibrosis within the scala tympani following implantation could act as a space-occupying foreign body that interferes with the natural mechanics of the cochlea [78]. These issues remain current. It should be acknowledged that some delayed hearing loss may reflect progression of the underlying cause of deafness and as such, cochlear implantation may cause an accelerated loss of residual hearing as a result of electrode insertion [79]. Even with meticulous surgical technique, insertion of the electrode through the cochleostomy is essentially a blind technique and some loss of residual hearing is almost a ubiquitous finding [62]. Whether cochlear implantation-related hearing loss is determined at the time of surgery, as a natural consequence of subsequent inflammation, apoptosis and tissue response to a foreign body, or whether the latter may be modulated by pharmacological intervention is under investigation in a number of laboratories.

Fibrosis within the cochlea and around the electrode array is an almost ubiquitous finding following implantation. This is not unique to cochlear implantation as fibrosis can occur as a reaction to other inflammatory processes to disrupt inner ear anatomy without electrode placement [80, 81]. It is common at the cochleostomy site, along the array and also extends beyond the tip of the electrode [45, 82]. Fibrosis appears to be worse along the point on the lateral wall where the electrode turns around the basal turn [45]. The presence of fibrosis along the basal turn is predicted to alter the vibration of the apical basilar membrane and thus interfere with residual low frequency acoustic hearing [83]. Surgical and pharmacological modifications that aim to reduce postoperative fibrosis within the cochlea are therefore important for hearing preservation during cochlear implantation, and their use may enable better understanding of the mechanisms behind this loss.

New bone formation within the cochlea is another commonly observed consequence of cochlear implantation. Osteogenesis typically occurs in similar locations to fibrosis: at the cochleostomy site, along the path of implantation, and even in the nonimplanted apex of the cochlea [79, 82].

Nonspecific inflammatory reactions within the cochlea have been known also to cause loss of residual hearing post-implantation [77]. Most reactions, however, are likely to be in response to the implant itself or to damage within the cochlea upon insertion. Following implantation, histological examination of the fibrosis at the basal turn suggests noninfected inflammatory cells, possibly indicating a foreign body reaction [84]. Closer characterisation of these cells types has shown a wide variety of inflammatory cells within the fibrotic tissue reaction, including mononuclear leukocytes, histiocytes, and foreign body giant cells [79].

Histological evidence suggests that there are many pathological processes associated with electrode insertion. It is unclear whether these pathological processes are associated with any observed hearing loss or cause the observed hearing loss. Direct electrode insertion trauma, apoptosis, acute inflammation, chronic inflammation and hypersensitivity/foreign body reaction can all potentially occur in the cochlea after implantation. With the common finding of inflammatory cells, fibrosis and giant cells, it could be postulated that potent anti-inflammatory drugs like corticosteroids have a role to play in hearing preservation. What remains unclear is what effect these pathological processes have on the hearing mechanisms of the cochlea, although it seems certain that the preservation of residual hearing through maintenance of neuronal survival and cochlear health can only be beneficial to the implant recipient.

## 4. Interventions to Promote Hair Cell and SGN Survival for EAS

As discussed above, hearing preservation and the survival of both residual hair cells and SGNs in cochlear implant surgery have become an increasingly important goal in order to facilitate EAS outcomes. Along with surgical refinement, the delivery of therapeutic agents to the cochlea has the potential to provide protective effects within the cochlea and is likely to have significant clinical benefits.

Various techniques, treatment regimes, and therapeutic agents are currently being used or are under investigatation for future use in this regard. Localised delivery of therapeutic agents into the cochlea is likely to be the most effective means to promote neuronal health, as it places the therapeutic agents in direct proximity to the target cells they are intended to protect. In addition, this approach minimises the high dose rates and other complications that can arise from systemic delivery.

Direct infusion into the fluid spaces of the cochlea can be achieved via a cochleostomy to insert, for example, a cannula attached to a reservoir containing the drugs of interest, a drug-coated electrode, or to implant cells expressing therapeutic agents. Each of these techniques has been demonstrated to successfully deliver the drug in question to the cochlea and be very efficacious for SGN protection [22, 85–89]; however, the surgical procedures involved can be traumatic within themselves and are likely to cause damage to any existing hearing. As such, in cases where hearing is intact and hair cell preservation is a primary aim, such intracochlear techniques are not suitable. For EAS patients, combining the drug delivery and implantation surgeries is the most efficient way of reducing cochlear trauma and is likely to become the protocol of choice in the future. Drug delivery to the inner ear can also be achieved via diffusion across the round window membrane. The round window membrane is known to be permeable to large molecules including neurotrophic factors, and indeed, the effects of neurotrophic factors have been detected in the perilymphatic fluids of the cochlea following application onto the round window via loading into alginate beads [90], hydrogel [91] or Gelfoam [92], and therapeutic effects on hair cells and SGNs were observed [91, 92]. Importantly, the surgical approach required for this method of application is minimally invasive and has a much lower risk of potential trauma to the hair cells and the patient's existing hearing. This delivery method could be used, for example, when an electrode array is already in place and drug delivery is required. While the delivery methods are important and significant clinical consideration is required for application in human recipients, this section will focus on the therapeutic agents themselves.

Several classes of compounds are available as therapeutic agents to protect the sensory hair cells and SGNs from trauma and degeneration, including neurotrophic factors, antioxidants, antiapoptotic agents, and anti-inflammatory steroids, and a number of companies are currently involved in the commercial development of novel otoprotective drugs. Treatment with these agents, in combination with surgical refinement such as hypothermia, may enhance the outcomes from EAS.

### 4.1. Neurotrophic Factors

Brain derived neurotrophic factor (BDNF) and neurotrophin-3 (NT3) are neurotrophins that are produced by the hair cells [93, 94] and supporting cells [95, 96] of the organ of Corti, and provide support to the SGNs during both development and adulthood. Loss of these endogenous neurotrophins, as occurs following a sensorineural hearing loss (SNHL), leads to SGN degeneration. However, it is now well known that exogenous application of neurotrophins can rescue SGNs from deafness-induced degeneration [85, 87, 97–101]. Importantly, we have recently reported long-term survival of SGNs following cell-based neurotrophin treatment in both the cat [88] and the guinea pig [86]. In addition, neurotrophic factors including glial cell-derived neurotrophic factor (GDNF), basic fibroblast growth factor (bFGF), and NT3 have been reported to protect hair cells from ototoxic drugs and noise damage [102–106]. Furthermore, auditory thresholds have also been decreased in normal hearing guinea pigs using BDNF diffused across the round window, a procedure that therefore has the potential to be used to protect residual hearing following cochlear implantation [107] and, thus, optimise EAS outcomes.

While the specific effects of neurotrophins have not been investigated in studies using EAS, enhanced SGN survival, and decreased electrically-evoked auditory brainstem response EABR thresholds were observed when neurotrophins such as BDNF were delivered in conjunction with chronic electrical stimulation from a cochlear implant [99, 108]. In EAS recipients, the remaining apical hair cells may also provide a source of neurotrophins for the SGNs in the damaged but electrically-stimulated regions, a prospect that warrants further investigation. Neurotrophins therefore hold great promise for use as protective therapeutic agents for both SGNs and hair cells, to facilitate and enhance the outcomes of EAS.

### 4.2. Anti-Inflammatory Steroidal Drugs

Corticosteroid treatments are now a common therapy for many forms of hearing loss, such as autoimmune inner ear disease, sudden hearing loss, and Menière's Disease [109]. More recently, numerous studies have demonstrated that the targeted delivery of steroids can protect hearing during cochlear implant surgery [49, 110–113]. The benefits of corticosteroids seen in these situations are mediated through both anti-inflammatory and antiapoptotic pathways [114]. The most common agent used in these studies is dexamethasone, and protection can be achieved by either pretreatment of the cochlea or postoperative infusion. Local delivery of corticosteroids is superior to systemic delivery, with the additional benefit of reducing any potentially harmful side effects that come with systemic administration [49, 110–113]. Pretreatment of the cochlea with dexamethasone applied onto the round window membrane can prevent the elevation of auditory thresholds that are typically associated with cochlear implantation and preserve the SGNs in the region of implantation [115–117]. A further study in cochlear implant recipients demonstrated that the combination of pre- and intraoperative glucocorticoids improved hearing preservation in adults and children with residual hearing [118]. In addition to the identification of dexamethasone as a potential therapeutic agent for protection of the inner ear against trauma, clinical trials using a novel sustained release delivery system for this drug, known as OTO-104, have reported positive phase 1 trial results [119], and phase IIb clinical trials have recently commenced.

### 4.3. Antioxidants

Numerous aetiologies of hearing loss occur as a result of the generation of reactive oxygen species and subsequent oxidative stress. For example, cisplatin, a potent antineoplastic agent used for the treatment of a variety of tumours and aminoglycoside antibiotics, which are commonly used to treat aerobic, gram-negative bacterial infections, has ototoxic side effects due to the generation of reactive oxygen species and oxidative stress [73, 120]. In addition, the formation of oxygen free radicals is hypothesised as a major cause of noise-induced hearing loss [104, 121]. Importantly, numerous studies have demonstrated that the application of various antioxidants, including n-acetyl cysteine, glutathione, resveratrol, and superoxide dismutase, can protect the auditory system from the degenerative effects of ototoxin- or noise-induced hearing loss [122–127]. Interestingly, therapeutic agents used for other clinical conditions, such as rasagiline, a monoamine oxidase type B inhibitor that is FDA-approved for use in Parkinson's disease, and metformin, an anti-diabetic drug, have also been demonstrated to attenuate hearing loss following ototoxin exposure [128, 129]. Clinical application of antioxidants for protection in the inner ear is also being developed: a molecule known as as SPI-1005, which induces glutathione peroxidase and reduces reactive oxygen species, is currently undergoing Phase II clinical trials [110].

### 4.4. Antiapoptotic Agents

Hearing loss has also been associated with the initiation of apoptotic pathways such as those mediated by c-Jun N-terminal kinase (JNK) and the caspases. The inhibition of each of these apoptotic proteins inhibits aminoglycoside-induced hair cell death [130, 131]. Furthermore, the induction of heat shock proteins (HSPs) in response to cell stress can significantly inhibit apoptosis in many systems. In particular, HSP70 has been shown to have protective effects against aminoglycoside-induced hearing loss and associated hair cell degeneration [132]. Clinical developments for the delivery of anti-apoptotic agents are also underway, with phase IIb clinical trials using the JNK inhibitor AM-111 reporting positive results including substantial improvements in hearing thresholds and speech discrimination scores.

### 4.5. Combinatorial Therapies

An effective treatment strategy to promote residual hearing via preservation of hair cells and SGNs may require a combined application of a number of these therapeutic agents, or agents with multiaction properties which can elicit neuroprotective, antiapoptotic, and antioxidant effects. Indeed, the combined application of neurotrophic factors and antioxidants has previously been shown to protect both SGNs and hair cells against ototoxin-induced damage [133, 134], with the effects significantly enhanced over that observed with the neurotrophic factor alone [134].

An alternative promising combined treatment option in the cochlea is the use of cell-based therapies, in particular, NTCELL, which are alginate encapsulated porcine choroid plexus cells (Living Cell Technologies Pty Ltd.) has potential application in the cochlea. Clinical trials for the treatment of Parkinson's Disease by NTCELL have recently begun. NTCELL secretes many neurotrophic and growth factors which can increase neuronal survival in response to traumatic injury, hypoxia or chemical challenges [135]. Specifically, NTCELL has been reported to secrete GDNF, BDNF and vascular endothelial growth factor (VEGF) in quantities sufficient for biological activity [135]. Furthermore, NTCELL also produces high levels of enzymes and proteins with antioxidant activities [135]. We recently reported that long-term implantation of NTCELL into the profoundly deaf cat cochlea promotes the survival of SGNs and their peripheral processes when combined with chronic electrical stimulation from a cochlear implant [88]. The stability and biocompatibility of NTCELL demonstrates the potential as a long-term technique for the delivery of therapeutic proteins to protect both hair cells and SGNs.

Future therapies may also incorporate the initial application of a steroid such as dexamethasone to protect against implant-related trauma, as well as an ongoing delivery method for neurotrophic factors and/or free radical scavengers for more long-term protective effects. Alternatively, gene therapy may facilitate long-term neurotrophic support to promote preservation of hearing.

## 5. Long-Term Protective Gene Expression via Gene Therapy

**Figure 4:**
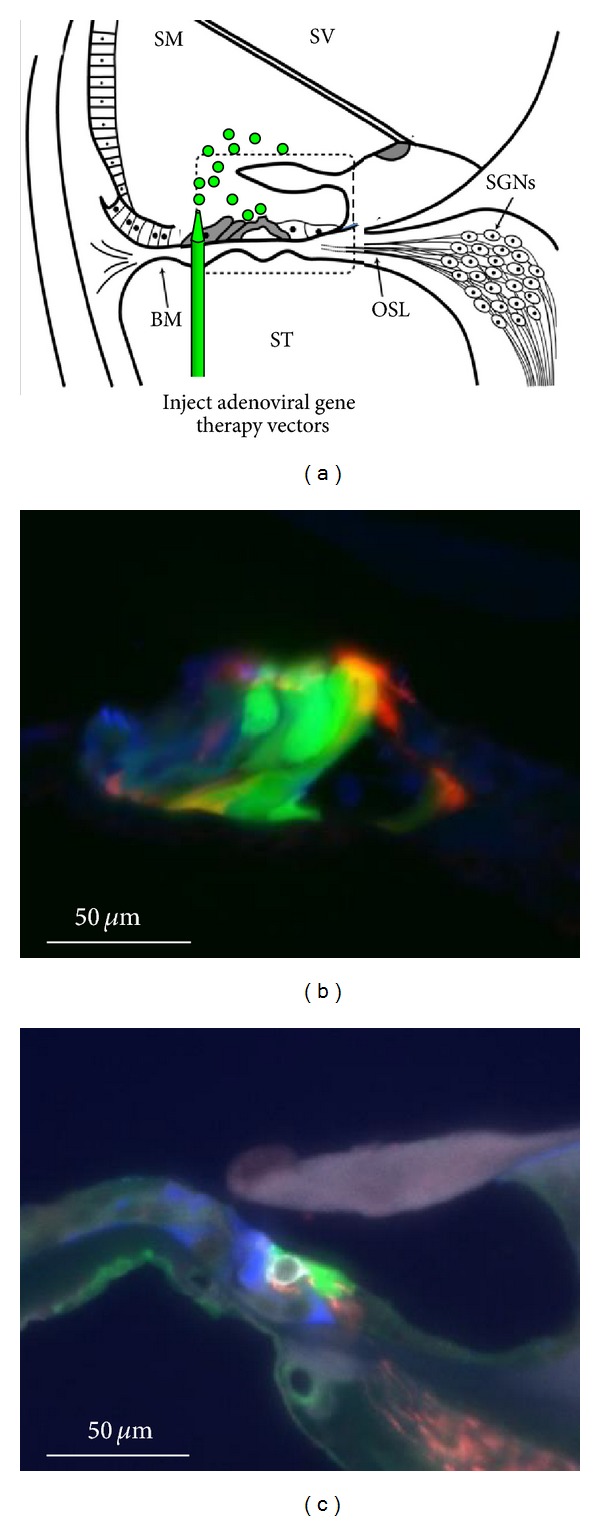
Gene therapy in the cochlea. (a) Injection of adenoviral vectors (green) carrying protective genes such as neurotrophic factors into the scala media of the cochlea results in gene expression in the organ of Corti (shaded cells) and enables protection of hair cells and SGNs. Dashed rectangle shows area of the cochlea shown in (b) and (c). (b) In the normal hearing cochlea, gene expression (green) can be observed in hair cells and supporting cells of the organ of Corti. Pillar cells are shown in red (phalloidin); other supporting cells are shown in blue (calretinin). (c) In a deafened guinea pig, the organ of Corti has degenerated at the time of gene therapy resulting in reduced gene expression (green). A degenerating hair cell is shown in white (myosin VIIa), supporting cells are shown in blue (calretinin) and nerve fibres are red (neurofilament heavy chain). SM: scala media; ST: scala tympani; SV: scala vestibuli; BM: basilar membrane; OSL: osseous spiral lamina; SGNs: spiral ganglion neurons.

The introduction of neurotrophins into the cochlea has proven to play a key role in the survival of SGNs after SNHL [20, 21, 101] and may play a role in the protection of hearing following cochlear implantation (see above). The finite source of neurotrophins in experimental (pump-based) delivery systems that eventually require replacement has led to increased interest in the use of gene therapy to maintain functional levels of acoustic hearing. Gene therapy has the potential to introduce neurotrophins into the cochlea with long-term gene expression arising from a single surgical intervention to the cochlea, potentially coinciding with cochlear implantation. Gene therapy with neurotrophic factor genes such as NT3, BDNF or GDNF resulted in long-term protection of SGNs after noise-induced, ototoxic, or hereditary hearing loss [100, 136–141]. From a single injection of adenoviral vectors containing neurotrophin genes into the scala tympani or scala media, there were at least 3 months of SGN protection after hearing loss [140, 142], with recent data indicating that adenovirus expression can extend to 6 months post injection [143]. However, a complicating factor in the long-term expression of transgenes in the cochlea is the degeneration of cells that are the potential targets. These include hair cells, supporting cells, and SGNs which can all degenerate after hearing loss (Figure 4).

Previous studies of neurotrophin gene expression, in supporting cells in particular, have demonstrated beneficial effects on SGNs such as survival and neuronal resprouting [100, 138] but did not prevent the supporting cells from degenerating at the same rate as those that were not expressing neurotrophins [140]. Eventually, the supporting cells and the neurotrophic transgene would be lost [143]. This study suggests that neurotrophin gene therapy needs to target cochlear cells that do not degenerate after hearing loss. Further studies have shown that injection of neurotrophin genes into the scala tympani resulted in expression in cells lining the perilymphatic space and protected SGNs after hearing loss [137, 142, 144]. Significantly, this is likely to be the situation for the preservation of residual hearing following cochlear implantation where the sensory and supporting cells are likely to be preserved at the time of implantation. Whether gene therapy can prevent loss of residual hearing after cochlear implantation is yet to be determined.

Similar to studies using other neurotrophin delivery systems, expression of BDNF by adenoviral vectors in combination with electrical stimulation from a cochlear implant promoted SGN survival and improved thresholds for SGN stimulation [142]. It is possible that concurrent electrical stimulation from the cochlear implant will affect the efficiency of the expression of the transgene and the efficacy of the neurotrophins produced, as previous studies have shown improved SGN survival when pump-delivered neurotrophins were combined with electrical stimulation [99].

### 5.1. Viral Vectors and Cell Specificity

Viral vectors are currently the most efficient way to introduce transgenes into cochlear cells. There are a number of viral vectors that have been tested in the cochlea including adenovirus, adeno-associated virus (AAV) and herpes simplex virus [145–148]. Each vector type has its unique cell specificity expression pattern in the cochlea (tropism), which can be exploited to improve the protective effects of gene therapy. For example, adenovirus type 5 has high tropism for supporting cells of the organ of Corti [149], hence, injection of adenovirus type 5 vectors carrying neurotrophin genes into the scala media was found to result in efficient transduction of supporting cells of the organ of Corti, in turn resulting in protection of SGNs after hearing loss [100]. AAV serotype 5 also has tropism for supporting cells [150], while AAV3 has specificity for inner hair cells [151] and herpes simplex virus targets neuronal cells [152]. There are currently over 100 serotypes of AAV and over 50 serotypes of adenovirus, each with different cell specificities, providing great potential to express transgenes in particular cell types. Cell specific promoters can also be used to achieve cell-specific gene expression, with the myosin VIIa promoter driving exclusive expression in inner hair cells as a striking example [153].

Cell-specific gene expression can have a big impact on the protective effects observed after hearing loss. Gene expression that was localised mainly to supporting cells of the organ of Corti not only had greater SGN survival compared to expression in cells of the scala tympani [100], but also may have a nerve guidance effect on the resprouting nerve fibres [100, 138]. Regenerating nerve fibres were highly disorganised when neurotrophins were introduced via a mini-osmotic pump to the scala tympani [101], but when adenoviral gene therapy was used to introduce neurotrophins into the scala media, nerve fibres were observed in greater density near cells expressing the neurotrophin genes, compared to the control GFP gene alone [100].

Expression of reporter genes in SGNs has been demonstrated after injection into the scala tympani using vectors such as AAV [151, 154] and adenovirus [155]. However, no studies have reported neurotrophic factor transgene expression within SGNs as a means to preserve SGNs, and this is an area for future research as a means to improve EAS outcomes through hearing protection.

### 5.2. Cochlear Injection Sites for Gene Therapy

The anatomy of the cochlea makes it suitable for localised gene delivery in many ways: It is surgically accessible; the fluid chambers are partitioned allowing certain cells to be targeted; and the blood-cochlear barrier ensures there is minimal spread of the virus beyond the injection site. The scala tympani is easily accessible via the round window membrane or a cochleostomy, similarly to the insertion route used during cochlear implantation. Injection of viral vectors into the scala tympani results in gene expression predominantly in cells lining the perilymphatic space, but also in hair cells and supporting cells [100, 156]. The scala media is much smaller, surrounded by tight junctions and is more difficult to access either by injection through the basilar membrane or through the lateral wall of the cochlea [100, 157]. Injection into this compartment results in more localised expression in hair cells, supporting cells and interdental cells [100, 156, 157]. Despite the complexity of the surgical approach to the scala media, expression of protective genes such as neurotrophins has a big impact on SGNs due to the proximity of supporting cells to the SGN nerve endings (see Figure 4) [100, 138]. Given that the target for gene therapy is the protection of residual hearing in the apical cochlear regions, delivery of the transgene to the scala tympani would be the best route to enable expression in the apical regions [100]. This also facilitates the administration of viral vectors at the time of cochlear implantation, meaning that only a single surgery is required for the two interventions, and the gene therapy can function from the moment that the electrode array is inserted.

In most cases, injection of viral vectors into the cochlea results in gene expression that is localised to the cochlea. However, there have been reports of viral vector spread to the contralateral ear as well as the cerebellum [158, 159]. Furthermore, the utricle, saccule, or semicircular canals of the vestibular system contain fluids that are continuous with cochlear perilymph and endolymph. Hence, injection into the cochlea also results in gene expression in the vestibular system in many cases [160, 161]. This connection can be exploited as an additional surgical injection site for gene expression in the cochlea, often avoiding the loss of hearing that accompany direct injection into the cochlea [161]. Injection into the cochlea or the vestibular system all require surgical intervention, but studies have shown that viral vectors carrying reporter genes or protective genes can cross the tympanic membrane of the cochlea and exert protective effects, at least on hair cells, without the need for surgery [162, 163].

### 5.3. Clinical Translation of Gene Therapy

It is important to demonstrate the clinical safety of gene therapy in the cochlea. Gene therapy is finding increased clinical acceptance with multiple clinical trials demonstrating that viral gene therapy in the human nervous system is both beneficial and safe. AAV in particular has been used in numerous clinical trials. For example, AAV2-GAD (glutamic acid decarboxylase) is being trialled for advanced Parkinson's disease and AAV2-RPE65 (retinal pigment epithelium-specific 65 kDa protein) is under investigation for severe retinal dystrophy. No serious adverse effects attributed to the vector were observed and each showed apparent biological effects of the transgene [164–166]. Of particular note is the long-term transgene expression observed from AAV vectors in clinical and preclinical trials, up to eight years in one case in nonhuman primates [167, 168]. In the cochlea, the main issues of viral vector gene delivery are the potential for immunological responses (inflammation) and toxicity. Modern AAV vectors have shown little evidence of toxicity in the cochlea and in particular bovine AAV was shown to be expressed in supporting cells and SGNs with no effect on hearing showing that it does not harm the delicate sensory cells of the cochlea [149]. For adenovirus, strong immune responses were reported for first generation adenoviral vectors [169, 170]. Unfortunately, when an advanced generation virus that lacks all viral coding sequences was introduced into the supporting cells of the cochlea, loss of hearing was still reported indicating enduring ototoxicity [149].

Although more controlled trials will be needed to prove the overall safety and effectiveness of gene therapy, it is encouraging that gene therapy has been conducted in the human nervous system. There is increasing evidence that gene therapy is providing clinical benefits to a range of diseases with ever improving vector design eliminating the adverse events that used to be associated with gene therapy [171]. A recent study by Pinyon et al. [172] used stimulation through a cochlear implant to cause localised electroporation of the mesenchymal cells in the perilynphatic canals in an animal model. This enabled focalised delivery of BDNF genes, and regrowth of SGN neurites close to the cochlear implant electrodes was observed. The combination of cochlear implantation with administration of gene therapies in the same surgery in recipients with low-frequency residual hearing is a compelling prospect. In this way, hearing would be restored (via the implant) and residual hearing would be both maintained and perhaps enhanced, from a single intervention. In order for this to become a reality, a method for long-term expression needs to be developed [173], as this is currently the missing piece to the EAS puzzle.

## 6. Conclusions

Despite being an emerging field, EAS promises to improve the lives of partially deaf people to whom a hearing aid does not provide a satisfactory listening experience.

There are two clear areas of research required to improve EAS outcomes: improving our understanding of how EAS is processed by the brain, and prevention of the hearing loss that occurs in a significant proportion of EAS listeners. This paper has focused on these two key areas and has shown that the interactions between acoustic and electrical stimulation at the level of the cochlea and along the auditory pathway require further exploration in order to understand their perceptual effects. This research, in turn, will lead to the refinement of processing strategies that will enable the benefits of combined EAS to be optimised to full effect, and minimise any interactions that may hinder ideal results.

The protection of residual hearing, both at the time of implantation and postimplantation, is of critical importance to successful EAS. A number of innovations have recently been made in terms of electrode array design and surgical technique that aim to promote residual hearing, but there are still reports of implantation-related hearing loss. As such, administration of protective agents prior to, during or after cochlear implantation could provide the required promotion of cochlear health to maintain residual hearing. There are a variety of therapeutic agents that have the potential to protect residual hearing and support SGN survival to enhance the benefits of EAS. Future studies in this field need to elucidate (i) the most suitable therapeutic agents, or combination thereof, (ii) the most effective treatment regime, which may be made up of various regimes and different drugs, and (iii) appropriate delivery methods, including acute administration and longer-lasting gene therapy treatments, in order to preserve residual hearing and SGN survival in cochlear implantation and maximise the benefits and outcomes of EAS.

We have shown that the combination of these agents with electrical stimulation can further promote neuronal growth [88] and it seems that this compelling combination deserves to be explored for the benefit of EAS recipients worldwide.
